# Inter-observer agreement according to malaria parasite density

**DOI:** 10.1186/1475-2875-12-335

**Published:** 2013-09-22

**Authors:** Mounkaila Abdou Billo, Mahamadou Diakité, Amagana Dolo, Mouctar Diallo, Belco Poudiougou, Sory Ibrahima Diawara, Eric S Johnson, Janet C Rice, Donald J Krogstad, Ogobara K Doumbo

**Affiliations:** 1 1512 Potomac Ave SE, Washington DC, USA; 2 Mali-Tulane Tropical Medicine Research Center, Malaria Research and Training Center, Faculty of Medicine, Pharmacy and Odontostomatology, University of Sciences, Techniques and Technology, P.O.Box 1805, Bamako, Mali

**Keywords:** Inter-observer agreement, Intra-class correlation, Kappa statistic, Parasitaemia, Thick smears, Microscopy

## Abstract

**Background:**

Recent developments in diagnostic techniques for malaria, particularly DNA probes and sero-immunology, have raised questions as to how these techniques might be used to facilitate malaria diagnosis at the most peripheral levels of the primary health care system. At present, malaria diagnosis is based on the standard microscopic examination of blood films in most field epidemiologic studies and is likely to remain so in the immediate future in Africa. The objective of this study was to assess inter-observer agreement for the examination of Giemsa-stained slides for *Plasmodium falciparum* parasites.

**Methods:**

Children aged 0 to 10 years were enrolled yearly in Bancoumana village (West Africa), mainly during the transmission season (June to October). The blood smears obtained from the persistently negative children in June 1996, August 1996, October 1996 and March 1997 were systematically re-examined. A stratified random sample (10%) proportional to the following parasite density classes 1–100, 101–5000, and 5001 and over was taken from the slides collected. The kappa statistics and the intra-class correlation were used as measures of agreement the first and the second slide examinations.

**Results:**

The weighted kappa statistic, widely used as a chance-corrected measure for nominal agreement, showed excellent inter-observer agreement (κ_w_=0.7926; 95% CI [0.7588, 0.8263]; *p*=0.01). The intra-class correlation co-efficient had the same value of 0.7926 confirming the appropriateness of the weighted kappa statistic. Inter-observer agreement for slides read as negative by one observer, or as containing more than 100 parasites per μl, was excellent: 97% (493/506) and 92% (145/158), respectively. In contrast, the inter-observer agreement for slides read by one observer as containing 1–100 parasites/μl was poor, 36% (96/268).

**Conclusions:**

In field conditions in Mali, there was a high reproducibility for slides reported as negative or as having more than 100 parasites per μl. However, smears with readings of 1–100 parasites per μl were less reproducible and should be re-examined carefully.

## Background

Measurement error is one of the major sources of bias in epidemiological studies. It can lead to spurious conclusions about the relationship between exposure and disease [1]. Recent developments in diagnostic techniques for malaria, particularly DNA probes and sero-immunology, have raised questions as to how these techniques might be used to facilitate malaria diagnosis at the most peripheral levels of the primary health care system [2]. At present, malaria diagnosis is based on the standard microscopic examination of blood film in most field epidemiologic studies and is likely to remain so in the immediate future.

Blood film examinations are crucial not only to distinguish parasitaemic from aparasitaemic children, but also to determine the parasite species and their density in the bloodstream. Thus, a correct reading will reduce misclassification bias and yield accurate effect measures.

The objective of this study was to assess the reproducibility of the results of thick blood smears obtained from a cohort of children of the village of Bancoumana, Mali (West Africa) by re-examining ~10% of the slides.

## Methods

The blood films were collected and prepared with the approval of both the Research Ethics Committees of the Faculty of Medicine, Pharmacy and Odontostomatology of the University of Bamako, Mali and Tulane University, New Orleans, USA. Study participants were enrolled yearly in Bancoumana village, mainly during the transmission season (June to October). All children aged up to ten years were included in the study. The malaria research and training centre has maintained a field laboratory in Bancoumana since June 1993. This village is located within a narrow riverine valley that has an area of approximately 10 sq km. The village itself consists of approximately 10,000 individuals living in 200 houses. Malaria occurs throughout the year with an average monthly prevalence of approximately 50%, with an intense seasonal transmission from June to November [3].

Thick smears were stained with 3% Giemsa (Sigma, St Louis, MO, USA) in phosphate buffer (pH 7.0) and examined using oil immersion magnification (1,000 X).

The blood smears obtained from the persistently negative children in June, August and October 1996 and March 1997 were systematically re-examined. Also, all negative slides during the four consecutive cross-sectional surveys (June 1997 to February 1998) were re-examined. In addition, a stratified random sample (10%) proportional to the following parasite density classes 1–100, 101–5,000, and 5001 and over was taken from the slides collected in June, August and October 1996 (see Table 1). Each slide was examined under oil-immersion (100 ×) until the microscopist had counted the number of asexual parasites (trophozoites) in fields containing 300 or more white blood cells. Parasite counts were estimated by multiplying the number of asexual parasites per 300 white cells by 25 (based on an average white blood cell count of 7,500 per μl) and expressed as the number of parasites per μl. At least 1,000 white blood cells were counted before a slide was recorded as negative. Slides were then re-examined by an experienced microscopist, blinded to the results of the first readings. Blood smears from the persistently negative children and those negative during the four cross-sectional surveys (June 1997 to February 1998) were re-examined. Of 7,550 thick blood smears, 932 (12.34%) were re-examined and classified by parasite density as follows: 0, 1–100 and >100 parasites/μl.

**Table 1:** Numbers of slides sampled and re-examined by parasite count category from June 1996 to March 1998

	**June 96**		**Aug. 96**		**Oct. 96**		**March 97**		**June 97**		**Aug. 97**		**Oct. 97**		**Feb. 98**		**March 98**	
Parasitaemia	S*	E**	S	E	S	E	S	E	S	E	S	E	S	E	S	E	S	E
0	136	120	136	110	136	120	134	112	20	20	20	19	20	20	20	19	0	0
1-100	54	48	32	27	22	13	34	29	35	35	29	28	22	20	26	26	35	35
101-5,000	40	39	40	37	40	37	0	0	0	0	0	0	0	0	0	0	0	0
5,001 +	6	6	6	6	6	6	0	0	0	0	0	0	0	0	0	0	0	0
Total	236	213	214	180	204	176	170	141	55	55	49	47	42	40	46	45	35	35

*Sampled.

**Located and re-examined.

The kappa statistics [4,5] and the intra-class correlation were used as measures of agreement the first and the second slide examinations [6]. In addition, Lin’s concordance correlation co-efficient for agreement [5] and the limits-of-agreement statistics and graphic procedures [6,7] complemented the aforementioned statistical measures of intra method reliability. The preliminary results showed that agreement is low among positive slides ≤100 per μl (32.2%) and very high among negative slides (97.4%). Therefore, only positive slides with a parasite density ≤100 per μl were systematically sampled during the remaining six cross-sectional surveys (March 1997 to February 1998).

A total of 932 slides out of 7,550 (12.34%) obtained from children of the nested case–control were reread, and 117 out of 1,049 (11.15%) slides sampled were not seen. Slides were re-read by an experienced microscopist, blinded to the results of the first readings. When the parasite density was ≤3 per 300 leukocytes, a second experienced microscopist re-examined the slide and an average count was reported.

## Results

Out of 7,550, 932 (12.34%) thick blood slides were re-examined by two well-trained microscopists to measure the reproducibility of the parasite density counts obtained during the cross-sectional surveys (Table 2).

**Table 2:** **Distribution of *Plasmodium falciparum *counts by readings**

**Second readings**					
**First readings**		**0**	**1-100**	**≥ 101**	**Total**
	**0**	493	1	12	506
	**1-100**	110	96	62	268
	**≥101**	5	8	145	158
	**Total**	608	105	219	932

When the measure of interest in a reliability study is an ordered categorical variable, such as the classification of *Plasmodium falciparum* density in this study, the weighted κ (κ_w_) is the appropriate measure [4]. The κ_w_ was calculated for the data presented in Table 2. The κ_w_ shows high agreement, with a result of 0.7926 (*p*<0.00001; 95% CI [0.7588, 0.8263]). The intra-class correlation co-efficient had the same value of 0.7926, confirming the appropriateness of the weighted kappa statistics [8]. The observed agreement among the negative slides was excellent, 97.43% (493/506). Conversely, the observed agreement for positive ≤100 was poor, 35.82% (96/268).

Collapsing the data in a 2×2 table according to the presence or absence of *P. falciparum* parasite in a thick blood smear gives a Cohen’s κ of 0.7179 (*p*<0.0001; 95% CI [0.6737, 0.7621]), indicating a excellent reproducibility between the first and the second readings.

**Figure 1:**
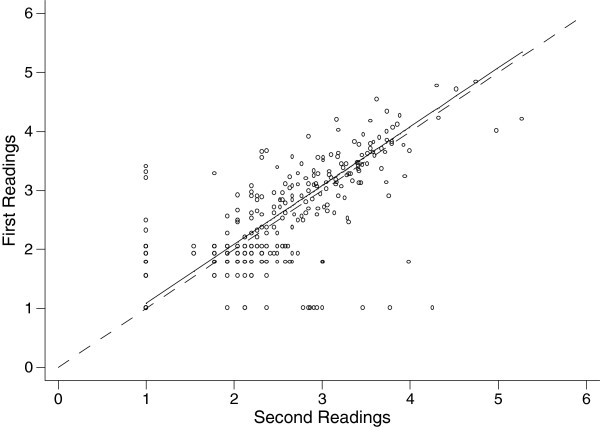
Transformed parasite counts of first and second readings, with line of perfect concordance and regression line using all data (932 slides).

**Figure 2:**
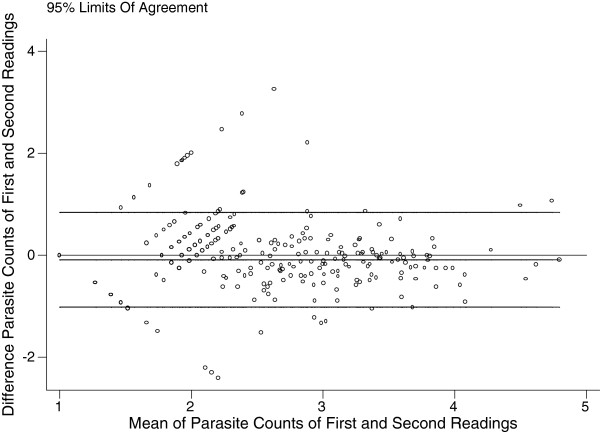
Difference against average of transformed parasite counts of first and second readings, with 95% limits of agreement using all data (932 slides).

Figure 1 shows the concordance correlation co-efficient [5] computed on the logarithm transformation of the parasite density using William’s method [lnpf = (pf+1)]. This correlation co-efficient [5] was 0.835, 95% CI (0.816-0.855) using logarithm-transformed parasite counts, and yielded a regression line with near-perfect concordance between the first and the second readings: an average difference of −0.088 ±0.474 [9] (Figure 2).

## Discussion

Currently, in many African countries, the accepted diagnostic technique for malaria is the examination of stained blood films under the oil immersion lens of the microscope. Serology and molecular technique play a part in epidemiology and in various special investigations [10,11]. Light microscopy has a central role in parasite identification and quantification and remains the main method of parasite-based diagnosis in clinic and hospital settings. Thick blood films allow a rapid examination of a relatively large volume of blood, enabling the detection of even scanty parasitaemia of all blood parasites. A well-prepared thick blood film gives more than a ten-fold increase in sensitivity over thin films [12].

Malaria prevalence is decreasing in many African countries. Therefore, the ability to identify all parasites becomes increasingly important. Good quality microscopy conducted by skilled technicians with capacity to manage appropriate quality control, and the currently available rapid diagnosis test (RDT), requiring less training than microscopy, are generally adequate for diagnosis in people who have acute malaria [12]. However, there are issues to be addressed with both procedures. Ensuring the quality of microscopy used for routine diagnosis has often proved difficult as the sensitivity and specificity of routine microscopy is significantly lower when compared with that of qualified microscopists based in reference laboratories [13]. This underlies the need for good training in microscopy for staff in remote areas. The choice of routine diagnosis of malaria in areas of low parasitaemia is microscopy, which is technically more difficult but is better for species identification and for estimating parasite densities, or diagnosis with the user-friendly RDT, which gives a positive or negative result (but not a measure of the density of parasites) and is not good for detecting *Plasmodium vivax* and the other non-falciparum parasites.

Parasite density estimation is highly valuable for the clinician, as it is an important determinant of treatment schedules for *P. falciparum*. If parasite density exceeds 10% in *P. falciparum*, exchange transfusion may be indicated [14,15]. A variety of studies have clearly demonstrated that microscopic diagnosis of malaria can vary greatly in its accuracy, particularly at low parasitaemia rates [12,16,17]. This variation in specificity and sensitivity is routinely observed in clinical settings, where a high proportion of reporting patients are parasitaemic and parasite densities are relatively high. In this study, the weighted kappa statistic, widely used as a chance-corrected measure for nominal agreement, showed excellent inter-observer agreement (κ_w_=0.7926; 95% CI [0.7588, 0.8263]; *p*=0.01). Inter-observer agreement for slides read as negative by one observer, or as containing more than 100 parasites per μl was excellent: 97% (493/506) and 92% (145/158), respectively. Dowling and Shute compared parasite counts obtained by examination of thin and thick smears and conducted that parasite losses of 60 to 90% occurred with thick films, whereas since thin films are fixed after drying and before staining, they assumed no significant loss of parasites during staining [18].

In a series of parasite dilutions, studies have found that thick films tended to measure parasite densities around one log lower than the number calculated to be in the dilution and this did not vary by microscopist [13,19]. O’Meara *et al.*[19] have shown that parasitaemia from the thick smear averaged 10% lower than the total mean (*p* = 0.001) and they have also shown that white blood cells were much less uniformly distributed that the parasites. They also confirmed that up to 60% of parasites were obscured in the thick film or lost during the process of red cell lyses and parasite staining. In this study, agreement was compared between two highly qualified microscopists according to parasite densities.

In contrast, the inter-observer agreement for slides read by one observer as containing 1–100 parasites/μl was poor, 36% (96/268). The concordance correlation co-efficient [5] was 0.835, 95% CI (0.816-0.855) using logarithm-transformed parasite counts, and yielded a regression line with near-perfect concordance between the first and the second readings: an average difference of −0.088 ±0.474 [10] [Figure 2]. Greenwood and Armstrong [20] have suggested that variation in parasite density depends in variability in the volume of blood used to prepare thick films being less than the variability in white blood cell count in the population they studied.

When two parasite counts for the same slide were compared, Killian *et al.* found considerable variability, with one reading being 0.12 to ten times the other [21]. They examined inter-rater variability in the results of malaria microscopy in epidemiological studies using 711 thick blood films re-read by four experienced microscopists. They also calculated parasite density by counting the number of trophozoites in 100 oil immersion fields and multiplying by four to give parasites per microlitre, assuming a blood volume of approximately 0.25 μl per 100 microscope fields. There was significantly less variability at parasite densities above 500/μl, 0.2 to 3.6 times. Overall, for variation between readers, O’Meara *et al.* stated that discrepancies in parasite densities reported by experienced clinic microscopists decreased with increasing mean density and trends were similar for *P. falciparum* and for *P. vivax* when they were considered separately [22]. When agreement between readers is required, it is important to apply an identical technique which seems to be more important than increasing the number of microscope fields read [22]. In another study, these authors found a significant inverse correlation between discrepancy among microscopists and mean parasite density [23]. Furthermore, they suggested that random chance in the selection of fields to examine may play a large part in reader discrepancy, especially with low parasitaemia. In a recent review, Makler *et al.* concluded that factors such as undertraining of microscopists, lack of microscopes and staining materials, and processing and reading large numbers of blood smears, dramatically increased the range for error [24]. Using the method described by Alexander *et al.*[25], similar findings were observed in the present study (see Additional file 1). When back-transformed to the original count, the limits with agreements increase with parasite density, and are much wider.

Since most elimination efforts will need to deal with both low parasitaemia and non-falciparum species, diagnosis becomes a major challenge for elimination programmes. Bowers *et al.* have shown differences between methods using the same microscopy staff, but reader technique itself clearly contributes to the accuracy of parasitaemia estimates [26]. Although the propensity of a gametocyte carrier to transmit infection is related to the density of gametocytaemia, individuals with very low gametocyte numbers can still transmit malaria infection and can be an important part of the reservoir of infection. Thus, elimination programmes will need to detect and treat all potential transmitters of infection with a more sensitive detection test. The slide readers in this study were all experienced malaria microscopists and the results may be different with less experienced readers. In the light of this and under low parasite prevalence, low parasite rates, and inadequate equipment conditions, for any parasite density less than 100 parasites/μl at least two experienced microscopists should blind read the slide.

## Conclusion

Improved means to detect asymptomatic persons with low parasitaemia will be crucial to malaria elimination. These results suggest a high reproducibility for slides reported as negative or as having more than 100 parasites per μl. However, low parasitaemia (<100 parasites/μl are less reproducible and should be re-examined carefully. In addition, a uniform counting protocol should be used and the number of white blood cells counted should be increased in order to improve inter-reader agreement. Until rapid, reproducible and quantitative PCR for malaria is widely available at low cost, microscopy will remain the method of choice for parasite density determination in malaria elimination phase as most African countries are observing a decrease in malaria prevalence.

## Competing interests

The authors have declared that they have no competing interests.

## Authors’ contributions

AD and MD were the reference microscopists; OKD was the trainer in malaria slide microscopy and served as the third reference microscopist for the entire Mali-Tulane TMRC grant. AD, MD and OKD participated in drafting the manuscript and supervised the collection of samples. MAB, MD, ESJ, JCR, OKD and DJK conceived the study and participated in its design. OKD and MAB supervised all aspects of the study carried out in Bancoumana and the drafting of the manuscript. MAB, JCR and ESJ performed all the statistical analysis for the study. All authors read and approved the final manuscript.

## Supplementary Material

Additional file 1 **Between-readers variation in asexual parasites counts.** Title: Variation between readers in asexual parasites counts using Alexander et al. (2010) methods. Description: When back-transformed to the original parasite density count, the limits with agreements increase with parasite density, and are much wider. Click here for file
